# Prevalence of Heart Failure and Adherence to Process Indicators: Which Socio-Demographic Determinants are Involved?

**DOI:** 10.3390/ijerph13020238

**Published:** 2016-02-19

**Authors:** Alessandra Buja, Giuliana Solinas, Modesta Visca, Bruno Federico, Rosa Gini, Vincenzo Baldo, Paolo Francesconi, Gino Sartor, Mariadonata Bellentani, Gianfranco Damiani

**Affiliations:** 1 Laboratory of Public Health and Population Studies, Department of Molecular Medicine, University of Padova, 35122 Padova, Italy; vincenzo.baldo@unipd.it; 2 Department of Biomedical Sciences, University of Sassari, 07100 Sassari, Italy; gsolinas@uniss.it; 3 Agenas, National Agency for Health Services, 00187 Rome, Italy; visca@agenas.it (M.V.); bellentani@agenas.it (M.B.); 4 Department of Human Sciences, Social and Health, University of Cassino and Southern Lazio, 03043 Cassino, Italy; b.federico@unicas.it; 5 Regional Health Agency of Tuscany, 50141 Firenze, Italy; rosa.gini@ars.toscana.it (R.G.); paolo.francesconi@ars.toscana.it (P.F.); 6 Faculty of Medicine, University of Padova, 35122 Padova, Italy; gino.sartor@studenti.unipd.it; 7 Department of Public Health, Università Cattolica del Sacro Cuore in Rome, 00168 Rome, Italy; gianfranco.damiani@unicatt.it

**Keywords:** primary health care, health care research, quality of care, inequalities

## Abstract

Interest in chronic conditions reflects their role as the first cause of death and disability in developed countries; improving the management of these conditions is a priority for health care services. The aim of this study was to establish which sociodemographic factors influence adherence to standards of care for chronic heart failure (CHF). A generalized multilevel structural equation model was developed and applied to a sample of patients with CHF obtained from administrative data flows in six Italian regions to ascertain any associations between adherence to standards of care for CHF and sociodemographic variables. Indicators of compliance were adherence to beta-blocker therapy (BB-A) and Angiotensin Convertin Enzime inhibitor/Angiotensin Receptor Blocker therapy (ACE-A), and creatinine and electrolyte testing (CNK-T). All indicators were computed over a one-year follow-up. Among a cohort of 24,997 patients, the BB-A rate was 40.4%, the ACE-A rate 61.1%, and the CNK-T rate 57.0%. Factors found associated with adherence were gender, age, and citizenship. Our study shows an inadequate adherence to standards of care for CHF, particularly associated with certain sociodemographic characteristics. This suggests the need to improve the role of primary care in managing this chronic condition. The measures considered only apply to patients with a reduced Left Ventricular Ejection Fraction, hence a limitation of this analysis is the lack of information on left ventricular ejection.

## 1. Introduction

The prevalence of non-communicable diseases [1] is rising and chronic conditions have become the leading cause of death and disability, thus coming to represent a primary concern for the health services. This is mainly due to the aging of the population, the survival of patients after acute events that leave disabling sequelae, and the availability of treatments that give patients a longer life expectancy. Many patients with chronic conditions are treated by primary care services, making good-quality chronic care a challenge. This is particularly true when general practitioners (GPs) fail to adopt evidence-based guidelines for managing chronic conditions, and when patients are unwilling or unable to follow their GP’s recommendations. In other words, a strong degree of adherence is essential, and this can only be achieved when both doctor and patient perceive that a treatment has more benefits than risks or costs. According to the World Health Organization (WHO) [2], it is important to study the phenomenon of adherence to drug treatment, and adherence to behavioral measures and follow-up procedures. It is also essential to analyze the determinants of adherence in order to identify population groups likely to be scarcely compliant with disease management programs. Adherence to appropriate chronic disease management programs is a multifaceted issue influenced by how health care is delivered and by patient-related factors (called *antecedents*) that may have an impact on an individual’s propensity to follow medical recommendations; some sociodemographic factors are among these antecedents [3]. Several studies have shown that age, ethnicity and formal education have an important influence on adherence to health professionals’ advice [4,5], for instance. While a number of authors focused on a particular sociodemographic dimension as a determinant of health care disparities, few studies [6,7] have analyzed how these variables interact in their association with adherence to a good-quality disease management program in a more complex scenario.

Among chronic conditions, chronic heart failure (CHF) is characterized by multiple relapses, with an expected one-year hospital readmission rate of more than 50%, and one-year mortality rates of 30% [8,9]. CHF is consequently the most common hospital discharge diagnosis of all chronic conditions, accounting for a substantial economic burden on national healthcare systems. In this light, improving the outpatient management of CHF has been suggested as a cornerstone for preventing frequent hospital readmissions and the related costs [10]. We found some studies that examined differences in adherence between different population groups [11,12], but such evidence needs to be confirmed, especially in the context of Southern Europe where the immigration phenomenon is more recent and less developed than in Northern Europe. The countries from which immigrants come are also different. European Statistics [13] reveal that the number of immigrants/1000 inhabitants is lower in Italy, Spain and Portugal compared with Northern European Countries such as the United Kingdom, Germany or Sweden. Most immigrants in Italy in 2013 came from Romania, Albania and Morocco whereas, for example, in Germany the majority of the immigrants are from Turkey, Poland and Italy. 

The aim of this study was to measure overall adherence to standards of care for CHF, considering a complex set of factors, including many individual sociodemographic characteristics of the patients, variability in GPs’ attitudes, and the effects of local health authorities’ stewardship in supporting and motivating adherence to evidence-based guidelines. 

## 2. Methods

### 2.1. Context

Italy is divided into 20 regions, and each regional government is responsible for fulfilling the objectives of a National Health Plan through Local Health Authorities (LHAs), which are organized into health districts (HDs) providing primary health care and community services. All residents registered with the National Health Service (be they Italians or regular immigrants) have a General Practitioner (GP) of their choice. GPs have a gate-keeping role and decide on exemptions from co-payment for drugs and diagnostic tests for any chronic conditions, as established by the Ministry of Health.

### 2.2. Data and Variables 

This was a retrospective cohort study. The methods used in this study have been explained in detail elsewhere [14]. Briefly, six Italian regions took part in the VALORE project, an initiative of the National Agency for Regional Health Systems, for the purpose of assessing quality of care for chronic diseases and the organization of primary health care services [5]. For organizational reasons, the regions participating in the VALORE project did not provide administrative data for their whole regional population, but only for specific geographical subareas. In each region, raw data were extracted from the local data files and sent via file transfer protocol (FTP) to a single data management center, after anonymizing the coded personal identifier. The dataset used in our analysis was generated automatically by processing administrative records. 

To identify cases of CHF we used the following administrative records: (a) hospital discharge records with one main and up to five secondary diagnoses coded using the International Classification of Diseases, Ninth Revision, Clinical Modification (ICD9CM); (b) disease-specific exemptions from co-payment for health care, coded using the ICD9CM; (c) the population registry containing demographic details (year of birth, gender). 

We only enrolled people registered with the Italian National Health System (all Italian citizens and regular immigrants, *i.e*., foreigners with a regular entry visa or residence permit) and we classified nationality as follows: Italians; immigrants from highly developed countries (HDC); and immigrants from high-migratory-pressure countries (HMPC).

To assess adherence to process indicators we used two administrative records:

1. Drug-dispensing records coded using the Anatomical Therapeutic Chemical (ATC) codes for drug classification (adopted by the WHO), excluding drugs administered during hospital stays.
2. Outpatient care database, which records visits to specialist physicians and diagnostic tests (without the results).

We have measured the percentage of patients who performed each of the indicators and, using factor analysis, we have constructed a latent variable named “adherence to process indicators of a correct follow up”, which captures the adherence to all these indicators. 

In each region, record linkage within and between data files was done deterministically using a coded personal identifier. 

The standards for CHF care were chosen from those identified and established by the relevant scientific associations. In particular, we chose three indicators that the Heart Failure Work Group [15] considers indicative of the quality of care for CHF in terms of improving outcomes for outpatients with CHF, *i.e.*, adherence to therapy with ACE inhibitors or angiotensin receptor blockers (ACE-A); adherence to therapy with beta-blockers (BB-A); monitoring of creatinine, Na and K (CNK-T) every six months. Adherence to therapy was estimated from the presence of at least two prescriptions during the year of follow-up, separated by an interval of at least 180 days. The three indicators were computed over a one-year follow-up (1 January 2009 to 31 December 2009) by linking the cohort of patients to the administrative databases recording prescriptions for drugs and diagnostic tests.

To overcome any selection bias that might undermine the validity of our results we excluded all patients who died or were lost to follow-up because this loss was associated, in our database, with both exposure (age group) and outcome (adherence to the process indicator).

The Charlson Index (CI) [16] was calculated by applying Charlson’s algorithm to the hospital discharge diagnoses recorded for a patient in the previous three years. This index has proved a valid and reliable method for measuring comorbidities for the purpose of clinical research and, although it was first developed and validated for hospitalized patients, it has since been adapted and validated for primary care and community populations too.

### 2.3. Statistical Analysis

The data were summarized as numbers (percentages) of subjects for categorical variables. 

Prevalence was estimated as the total number of existing cases divided by the number of subjects in the sample, which corresponds to the whole resident population registered in the selected geographical subareas and alive at the index date. A standardized automated routine was developed in Stata 9.2 to apply a case ascertainment algorithm developed by the Tuscany Regional Public Health Agency, based either on diagnoses (both primary and secondary) in hospital discharge records, or on disease-specific healthcare co-payment exemptions [14]. In Italy, exemption is granted to patients in New York Heart Association stages III or IV. CHF recorded in hospital discharge records as a primary diagnosis implies that this condition was the main reason for admission. Its listing as a secondary diagnosis implies that it existed already at the time of admission or developed during the hospital stay and consequently influenced the treatment received and/or the duration of the hospital stay; this presumably happens mainly in cases with NYHA stages III or IV. The 95% confidence intervals (CIs) were calculated using a binomial distribution.

The chi-square statistic was used to test the hypothesis of independence between sociodemographic and clinical factors and adherence to standards of care. 

The structure of our dataset is hierarchical: individuals at level 1 are nested in their GPs (level 2), and the GPs are nested in the HDs (level 3). We thus consider the HD level and GP level as well as the individual level in a generalized three-level structural equation model (M-GSEM). To create the latent variable “adherence to evidence-based quality of care for CHF patients”, we hypothesized a statistical model in the form of a confirmatory factor analysis (CFA) defining the connections between the three quality-of-care indicators considered as binary variables [17]. The three variables are associated with their latent variable and this is represented graphically by arrows pointing from the constructs towards the indicator variable. Then an integrated generalized SEM model on three levels was formulated for the adherence outcome, considering the associations of the exogenous variables with the three indicators of the quality of care for CHF. Age group (reference 65- to 74-year-olds), gender (reference male), nationality (reference Italian), time since diagnosis (dichotomized as ≤3 years and >3 years), and CI (dichotomized as high/medium *vs.* low comorbidity) were the exogenous variables on the first level of the G-SEM model, each GP’s identification number was on the second level, and each HD’s identification number on the third. 

### 2.4. Ethical Considerations

The data analysis was performed on anonymized aggregate data with no chance of individuals being identifiable. The study complied with the Declaration of Helsinki and with Italian Law n. 196/2003 on the protection of personal data. The recent resolution n. 85/2012 of the Italian Guarantor for the Protection of Personal Data also confirmed the allowability of processing personal data for medical, biomedical and epidemiological research, and that data concerning health status may be used in aggregate form in scientific studies. Permission to use unidentifiable individual data extracted from administrative databases for the VALORE project was granted by ULSS 16 Padova, the ASP 7 Ragusa, the Assessorato Politiche per la Salute Emilia Romagna, and the Zona Territoriale Senigallia, which are responsible for the use of the data concerning their respective populations. The Agenzia Regionale di Sanità della Toscana is allowed by a regional law to use Tuscan data for research purposes. Approval for use of encrypted and aggregate data from the HSD was also obtained from the Italian College of General Practitioners.

## 3. Results 

The number of individuals aged 16 or more on 1 January 2009 amounted to 1,948,622 (930,891 Men *vs.* 10,177,319 women; 1,710,780 Italians). Among them, 16- to 44-year-old subjects were 42% (823,935) of the sample population, whereas +85-year-olds were the least represented age category (4%, only = 78,019). 

The algorithm identified 28,062 patients as having CHF, and 24,997 of these patients completed the one-year follow-up period. Table 1 shows the characteristics of the sample. 

The population rate of adherence for BB-A was 40.4%, for ACE-A it was 61.1%, and for CNK-T it was 57.0%. 

Table 2 shows the bivariate association between adherence to quality-of-care indicators and sociodemographic characteristics. All sociodemographic variables are significantly associated with process indicators in the bivariate analysis, except for nationality and BB-A.

**Figure 1:**
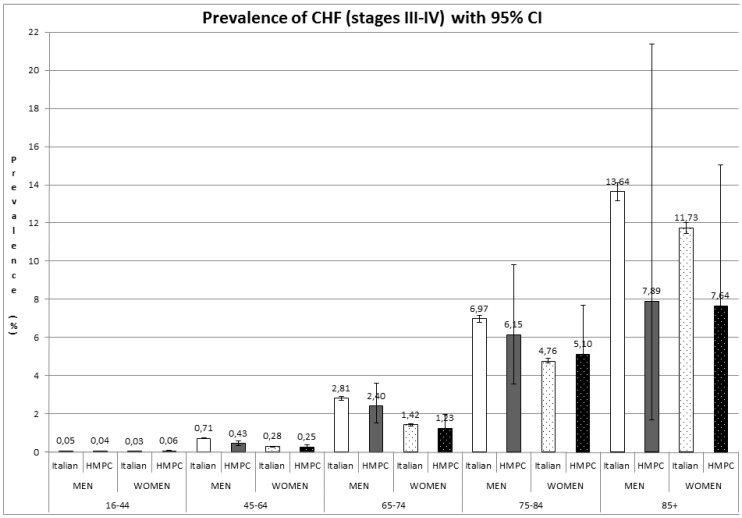
Prevalence of CHF (mainly stage III–IV NYHA) with 95% CI. HMPC: High-Migratory-Pressure Countries.

The estimated overall prevalence of CHF in the general population over 16 years old was 1.44% (95% CI: 1.42–1.46). Figure 1 shows the prevalence of CHF for men and women, respectively, by age group and nationality. 

Table 3 shows which variables are associated with adherence to process indicators in a multilevel structural equation model. The women category is associated with a lower adherence to therapy with ACE-I/ARB and BB whereas they seem more adherent to CNK testing. Younger patients (16–44) tend to adhere less both to therapy and testing than the reference age category (64–75). This is also true for patients 85+. The 45–64 category is instead associated with a higher adherence to BB therapy in respect to the reference. And also 75- to 84-year-old patients have their creatinine and electrolytes tested more. HMPC immigrants have lower adherence to all indicators compared to Italians. Patients with more comorbidities (high CI) are more adherent to BB therapy and CNK testing than patients with no/low comorbidity, but less adherent to ACE-I therapy. Patients with a more recent diagnosis are more adherent to ACE-I therapy than patients with no or low comorbidity. 

**Figure 2:**
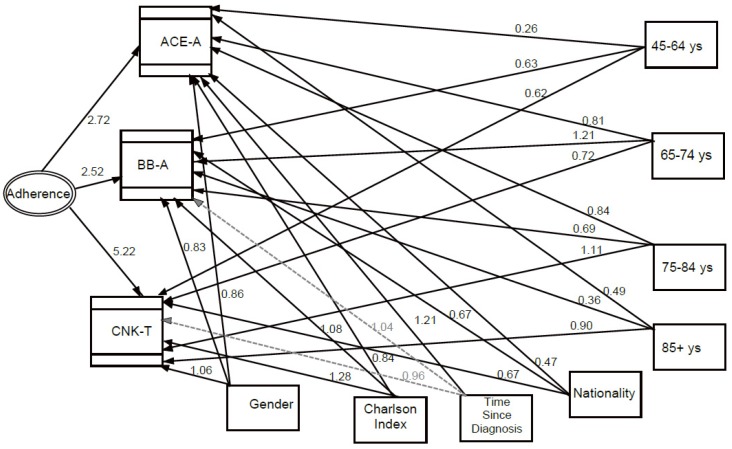
Multilevel structural equation model (SEM) with estimates of the parameters expressed as exponential functions of the regression coefficients (odds ratios). Direct effects are represented. Dashed grey lines stand for non-significant odds ratio paths, solid lines for significant odds ratio paths. (ACE-A = Therapy with ace-inhibitors or angiotensin-receptor blockers; BB-A = Therapy with Beta-Blockers; CNK-T = Creatinine, Sodium and Potassium Test).

The parameter estimates of the multilevel structural equation model (SEM) are also outlined in Figure 2, showing the association of each single process variable with adherence (the latent variable of the model), and it also illustrates the associations between single process indicators and sociodemographic characteristics.

## 4. Discussion

This population-based Italian study estimated the prevalence of CHF in the Italian and immigrant population by means of an administrative database record linkage. It revealed a suboptimal quality of CHF patient care, and disparities in their management, to the detriment of women and HMPC migrants.

The estimated prevalence of CHF among individuals aged 16 or more refers mainly to cases in the symptomatic stage of the disease, since the administrative data considered here concern co-payment exemptions and hospital discharge records involving a diagnosis of CHF. A validation study found that querying the clinical databases of Italian GPs produces similar prevalence estimates [14], which also resemble those reported in other European population-based studies [18] using screening methods, although the populations investigated differed in age of enrolment and the methods used to identify cases of CHF. The latter studies suggested a prevalence of CHF in the range of 1% to 2% [19]. 

Our data on the Italian population showed a higher prevalence in males than in females for all age groups (1.59% in Italian men and 1.5% in Italian women). The MONICA study [20] reported that the prevalence of definite Left Ventricular Sistolic Disfunction (ejection fraction of 30% or less) was 4% in men and 2% in women between 25 and 74 years of age. 

Our results also indicated that immigrants from HMPC generally have a lower prevalence of CHF than native Italians. Our data only showed a statistically significant difference for 45- to 64-year-old males, however. To our knowledge, no estimates of the prevalence of CHF among immigrants are available in the literature, but if we consider the broader category of heart disease, several studies have reported that immigrants have lower mortality rates of heart disease than native populations [21], which are findings consistent with the “healthy migrant” effect already seen for other diseases [22].

Like most other chronic conditions, the most important factor influencing the prevalence of CHF is age: like most studies conducted in North America [23] and Europe [24], our results reveal an exponential increase in its prevalence with age for both men and women, and for both Italians and immigrants. 

Overall, our data on adherence show a poor quality of care for all the CHF management indicators investigated, considering the thresholds of the Quality and Outcomes Framework (QOF) indicators in England [25] and the standards defined by the Italian Society of General Medicine (SIMG) [26]. A review found that the prevalence of ACEI/ARB use in 2001 ranged from 43% to 90% (median 71%) among patients discharged from the hospital with a known systolic dysfunction, and from 67% to 95% (median 86%) for those monitored at specialty clinics [27]. The findings of an Austrian study [28] on discharged patients (65% ACEI/ARB, 40% BB) are more similar to ours. The databases of the UK General Practice Extraction Service show instead that the average percentage of adherence to ACEI/ARB therapy or dual therapy with the addition of BB in England reaches proportions of 99% and 92%, respectively. These data only refer to patients without “exceptions” (e.g., patients or caregivers refusing the treatment, patients canceling or failing to attend appointments, or ignoring a GP’s advice), whereas the adherence rate for the same indicators drops to 88.31% and 77.10%, respectively, when all patients (including exceptions) were counted. Although these data might be comparable with ours (which include all patients), they still go to show that CHF patient management is of poorer quality in Italy than in the UK. Our data are consistent with smaller, regional studies [29] conducted in northeastern Italy, which reported adherence rates of 72% for ACEI/ARB therapy, and 46% for BB. 

In this work, we applied a generalized three-level SEM, hypothesizing an association between adherence and sociodemographic characteristics directly linked to four indicators of quality of care for CHF adopted in the guidelines, including an individual patient-level factor, a GP-level factor, and a HD-level factor, to account for variability in adherence on different levels. Sociodemographic factors had more than one significant path, being associated with almost all quality-of-care indicators. First, we found that women are less adherent to all indicators except creatinine and electrolyte monitoring.  One of the reasons for this could lie in the different etiology of CHF by gender: the condition develops after an acute myocardial infarction more often in men than in women, and this acute event frightens patients and alarms their GPs, making both of them pay more attention to the management of the condition. Women often develop CHF after a long history of hypertension without an acute event that might draw their attention to the problem. This theory is confirmed by the fact that women are more compliant than men in the case of other diseases, such as diabetes [30], when there is no substantial gender-related difference in the natural history of the condition. 

Our results also show that the clinical management of CHF generally remains suboptimal in the oldest and youngest age groups, *i.e.*, people who may be less able or too busy to contact primary care services. This gives rise to a higher risk of complications, rapid disease progression, and rising costs, despite the availability of effective treatments. 

Finally, our data indicate that being an immigrant reduces the likelihood of an appropriate adherence to therapy, the odds being 53% lower for ACEI/ARB and 33% lower for BB compared to the native Italian patients. Other studies in the US [12] show a lower adherence to therapy for CHF among migrant patients too. Migrants may experience language difficulties, issues concerning their cultural beliefs, and problems with obtaining transportation and/or time off work. They experience cultural barriers to health care despite the fact that there are no financial barriers in Italy, because chronically ill patients are exempted from charges for routine tests and drug prescriptions. Migrants who have arrived only recently may also be unfamiliar with how the health care system works in their new place of residence.

The WHO stated that it is essential to reorient and strengthen health systems to enable them to respond more effectively and equitably to the healthcare needs of people with chronic diseases (action plan). The emphasis is placed on diseases that affect women in particular, with a view to promoting women’s health and gender equity [31]. Our findings are alarming because an inadequate management of heart failure and of chronic heart conditions in general leads to higher costs, complications and worse health outcomes for patients. The Italian health system still does not focus strongly enough on primary care as a way to improve the longitudinal, comprehensive, coordinated and integrated management of chronic conditions, and consequently contain the acute events that raise health care costs and impair patients’ quality of life.

Judging from our findings, action is needed to reduce disparities, probably not (or not only) by means of strategies targeting specific disadvantaged patients, such as immigrants and the elderly, but (also) by devising a standard approach to the management of chronic conditions, and taking a more proactive stance on primary care. An empowerment philosophy should also include interactive teaching strategies designed to involve patients more actively in their own health care, addressing their cultural and social needs. Making patients more responsible for their day-to-day health care [30] could help to reduce all kinds of disparities, also assuring more disadvantaged patients timely access to effective services. Developing specific disease management programs, involving a figure who becomes responsible for all aspects of the patient’s care, strengthening the relationship between doctor and patient, and adopting a pay-for-performance system in which part of a GPs’ salary is based on their ability to prescribe appropriately and achieve health goals (also in terms of ensuring patients’ adherence to therapies) could be an effective organizational strategy for a better-quality management of chronic conditions. For instance, Doran *et al.,* [32] examined the relationship between socio-economic inequalities and the quality of the clinical care delivered in the first three years of the P4P scheme in England. They concluded that financial incentives have the potential to make a substantial contribution to reducing inequalities in the delivery of clinical care related to area deprivation. It has also been underscored that a multidisciplinary approach to managing CHF can improve patients’ clinical management and reduces the hospitalization rate and the related costs [33].

Our study has some limitations, primarily relating to the fact that not all the relevant socio-economic factors (such as level of formal education) were available in the database. The strength of our study lies in the fact that it was conducted on an unrestricted and unselected population of primary care patients, thus enabling an estimation of the prevalence of the disease of interest and of the related primary care performance measures. On the other hand, these data might be biased due to an opportunistic sample of LHAs being enrolled by the regional systems. This important methodological issue was addressed in a recent paper on the consistency of the VALORE database [14] used in the present study *vis-à-vis* other sources of data, such as primary care medical records and national surveys: in the case of CHF, the VALORE prevalence estimates were systematically higher than GPs’ estimates in all five regions considered, the largest difference being 1.4% *vs.* 1.1%. Another limitation lies in that we cannot know from our data whether the patient has a CHF with a reduced or preserved ejection fraction. This might lead to an overestimation of non-adherence to therapy indicators since ACE inhibitors and beta-blockers are mostly indicated for CHF with a reduction of the ejection fraction. Our study also focused on adherence to standards of care, as assessed by means of an administrative database, so our findings could be associated not only with the quality of care provided by physicians, but also with patient compliance. Adherence is a multifaceted behavioral issue, however, influenced by how health care is delivered by health care providers, as well as by patient-related factors. It is nonetheless a physician’s duty not only to prescribe appropriate therapies, but also to monitor patients and help them adhere to their prescriptions. Moreover, there is a difference in validity in data about drugs or diagnostic tests. Drugs appear in this record if they have been given to the patient by the pharmacists. We are then sure that the patient has collected his drugs, but not if he has swallowed them. However, we have considered patients to be compliant only if they had collected drugs at least twice during the year of observation, and it is likely that if someone buys the drugs twice in two different occasions, it is because he has finished the previous box. Instead visits and diagnostic tests appear in this database only if they are performed. Finally, the strength of our statistical analysis—as compared with a classical multivariate (logistic regression) method—lies in that: (1) we investigated the direct effects of socio-economic factors on adherence, measured as a construct variable using a model that realistically reflected the four quality-of-care indicators considered in the CHF management guidelines; (2) we regressed the latent variable of adherence between levels to control for the variability in adherence. Our application of this “innovative” data management model should encourage other researchers to use this novel methodological approach more frequently in the analysis of complex data. 

## 5. Conclusions 

Creative solutions are needed to address the escalating health care burden of chronic diseases. Adopting evidence-based approaches for the management of chronic conditions such as CHF can make health care systems more coherent and efficient, and provide a way to improve quality of care across a range of chronic health problems, as well as ensuring that primary care really is a service that comes as close as possible to where people live and work, with a level of care that guarantees fewer health disparities across population subgroups [3]. More action is still needed to promote a proactive, integrated approach to chronic care capable of actively involving chronically ill patients in all seasons of their life.

## Figures and Tables

**Table 1:** Characteristics of the sample.

Variable	Modality	Absolute Frequency	% Relative Frequency
Gender	Men	12,394	49.58
	Women	12,603	50.42
Age	16–44	303	1.21
	45–64	2765	11.06
	65–74	4891	19.57
	75–84	9442	37.77
	85+	7596	30.39
Nationality	Italian	23,606	94.45
	HMPC ^1^	1391	5.55
Charlson index	No comorbidity	6705	26.82
	Low comorbidity	6267	25.07
	High comorbidity	12,025	48.11
Time since diagnosis	<3 years	10,592	42.37
	>3 years	14,405	57.63

^1^ High-Migratory-Pressure Countries.

**Table 2:** Bivariate association (chi-squared test) between adherence to standards of care and sociodemographic characteristics.

Variable		ACE-A	*p*	BB-A	*p*	CNK-T	*p*
Gender	Men	7927 (63.96%)	0.000	5610 (45.26%)	0.000	6985 (56.36%)	0.000
	Women	7337 (58.22%)		4497 (35.68%)		7273 (57.71%)	
Age	16–44	110 (36.3%)	0.000	116 (38.28%)		130 (42.90%)	0.000
	45–64	1788 (64.67%)		1541 (55.73%)		1340 (48.46%)	
	65–74	3363 (68.76%)		2510 (51.32%)	0.000	2821 (57.68%)	
	75–84	6099 (64.59%)		3924 (41.56%)		5731 (60.70%)	
	85+	3904 (51.4%)		2016 (26.54%)		4236 (55.77%)	
Nationality	Italians	14,530 (51.55%)	0.000	9640 (40.84%)	0.540	13,500 (57.19%)	0.000
	HMPC	86 (43.22%)		77 (38.69%)		86 (43.22%)	
Charlson index	No comorbidity	4215 (62.86%)	0.000	2494 (37.20%)		3322 (49.55%)	0.000
	Low Comorbidity	4038 (64.43%)		2702 (43.11%)	0.000	3576 (57.06%)	
	High Comorbidity	7011 (58.30%)		4911 (40.84%)		7360 (61.21%)	
Time since diagnosis	<3 years	6376 (60.20%)	0.016	4137 (39.06%)	0.000	5763 (54.41%)	0.000
	>3 years	8888 (61.70%)		5970 (41.44%)		8495 (58.97%)	

**Table 3:** Results of the multilevel structural equation model with adherence as a latent variable; 95% confidence intervals (CI), odds ratios and *p*-values for adherence to process indicators.

Indicator	Variable	Modality	OR	95% CI		
				95% UL	95% LL	*p*
ACE-A	Gender (ref. Men)	Women	0.86	0.82	0.91	<0.001
	Age (ref. 65–74 years)	16–44	0.26	0.20	0.34	<0.001
		45–64	0.81	0.73	0.90	<0.001
		75–84	0.84	0.78	0.91	<0.001
		85+	0.49	0.46	0.54	<0.001
	Nationality (ref. Italians)	HMPC	0.47	0.35	0.63	<0.001
	Charlson Index (ref. No/low comorbidity)	high comorbidity	0.85	0.81	0.88	<0.001
	Time since diagnosis (ref. >3 years)	≤3 years	1.21	1.14	1.29	<0.001
BB-A	Gender (ref. Men)	Women	0.83	0.79	0.88	<0.001
	Age (ref. 65–74 years)	16–44	0.64	0.50	0.82	<0.001
		45–64	1.21	1.09	1.33	<0.001
		75–84	0.69	0.64	0.74	<0.001
		85+	0.36	0.33	0.39	<0.001
	Nationality (ref. Italians)	HMPC	0.67	0.50	0.90	0.008
	Charlson Index (ref. No/low comorbidity)	high comorbidity	1.08	1.04	1.12	<0.001
	Time since diagnosis (ref. >3 years)	≤3 years	1.04	0.97	1.10	0.270
CNK-T	Gender (ref. Men)	Women	1.06	1.01	1.13	0.029
	Age (ref. 65–74 years)	16–44	0.63	0.49	0.81	<0.001
		45–64	0.71	0.65	0.79	<0.001
		75–84	1.11	1.03	1.20	0.007
		85+	0.90	0.83	0.98	0.010
	Nationality (ref Italians)	HMPC	0.66	0.49	0.90	0.007
	Charlson Index (ref. No/low comorbidity)	high comorbidity	1.30	1.25	1.34	<0.001
	Time since diagnosis (ref. >3 years)	≤3 years	0.96	0.90	1.02	0.223

## References

[1] Bloom D.E., Cafiero E.T., Jané-Llopis E., Abrahams-Gessel S., Bloom L.R., Fathima S., Feigl A.B., Gaziano T., Mowafi M., Pandya A. (2011). The Global Economic Burden of Noncommunicable Diseases.

[2] De Geest S., Sabaté E. (2003). Adherence to long-term therapies: Evidence for action. Eur. J. Cardiovasc. Nurs..

[3] Buja A., Damiani G., Gini R., Visca M., Federico B., Donato D. (2014). Systematic age-related differences in chronic disease management in a population-based cohort study: A new paradigm of primary care is required. PLoS ONE.

[4] Mathes T., Jaschinski T., Pieper D. (2014). Adherence influencing factors—A systematic review of systematic reviews. Arch. Public Health.

[5] Agenas Valore Project Final Report. Evaluation of Management Models in Primary Health Care. https://www.ars.toscana.it/files/progetti/malattie_croniche/Relaz_finale_progetto_valore.pdf.

[6] Endo N., Goto A., Suzuki T., Matsuda S., Yasumura S. (2015). Factors associated with enrollment and adherence in outpatient cardiac rehabilitation in Japan. J. Cardiopulm. Rehabil. Prev..

[7] Bisiani M.A., Jurgens C.Y. (2015). Do collaborative case management models decrease hospital readmission rates among high-risk patients?. Prof. Case Manag..

[8] Kosiborod M., Lichtman J.H., Heidenreich P.A., Normand S.L., Wang Y., Brass L.M., Krumholz H.M. (2006). National trends in outcomes among elderly patients with heart failure. Am. J. Med..

[9] Rathore S.S., Masoudi F.A., Wang Y., Curtis J.P., Foody J.M., Havranek E.P., Krumholz H.M. (2006). Socioeconomic status, treatment, and outcomes among elderly patients hospitalized with heart failure: Findings from the National Heart Failure Project. Am. Heart J..

[10] Oddone E.Z., Weinberger M., Horner M., Mengel C., Goldstein F., Ginier P., Smith D., Huey J., Farber N.J., Asch D.A. (1996). Classifying general medicine readmissions. Are they preventable? Veterans Affairs Cooperative Studies in Health Services Group on Primary Care and Hospital Readmissions. J. Gen. Intern. Med..

[11] Frankenstein L., Clark A.L., Ribeiro J.P. (2012). Influence of sex on treatment and outcome in chronic heart failure. Cardiovasc. Ther..

[12] Zhang Y., Baik S.H. (2014). Race/ethnicity, disability, and medication adherence among medicare beneficiaries with heart failure. J. Gen. Intern. Med..

[13] Eurostat Statistics Explained—Migration and Migrant Population Statistics. http://ec.europa.eu/eurostat/statistics-explained/index.php?title=Migration_and_migrant_population_statistics&oldid=244940.

[14] Gini R., Francesconi P., Mazzaglia G., Cricelli I., Pasqua A., Gallina P. (2013). Chronic disease prevalence from Italian administrative databases in the VALORE project: A validation through comparison of population estimates with general practice databases and national survey. BMC Public Health.

[15] Yancy C.W., Jessup M., Bozkurt B., Butler J., Casey D.E., Drazner M.H., Fonarow G.C., Geraci S.A., Horwich T., Januzzi J.L. (2013). 2013 ACCF/AHA guideline for the management of heart failure: A report of the American College of Cardiology Foundation/American Heart Association Task Force on Practice Guidelines. J. Am. Coll. Cardiol..

[16] Charlson M.E., Charlson R.E., Peterson J.C., Marinopoulos S.S., Briggs W.M., Hollenberg J.P. (2008). The Charlson comorbidity index is adapted to predict costs of chronic disease in primary care patients. J. Clin. Epidemiol..

[17] Loehlin J.C. (2004). Latent Variable Models: An Introduction to Factor, Path, and Structural Analysis.

[18] Guha K., McDonagh T. (2013). Heart failure epidemiology: European perspective. Curr. Cardiol. Rev..

[19] Mehta P.A., Cowie M.R. (2006). Gender and heart failure: A population perspective. Heart.

[20] McDonagh T.A., Morrison C.E., Lawrence A., Ford I., Tunstall-Pedoe H., McMurray J.J. (1997). Symptomatic and asymptomatic left-ventricular systolic dysfunction in an urban population. Lancet.

[21] Sheth T., Nair C., Nargundkar M., Anand S., Yusuf S. (1999). Cardiovascular and cancer mortality among Canadians of European, South Asian and Chinese origin from 1979 to 1993: An analysis of 1.2 million deaths. CMAJ.

[22] Domnich A., Amicizia D., Panatto D., Signori A., Perelli V., Adamoli S., Riboli E.B., Gasparini R. (2013). Use of different subjective health indicators to assess health inequalities in an urban immigrant population in north-western Italy: A cross-sectional study. BMC Public Health.

[23] Redfield M.M., Jacobsen S.J., Burnett J.C., Mahoney D.W., Bailey K.R., Rodeheffer R.J. (2003). Burden of systolic and diastolic ventricular dysfunction in the community: Appreciating the scope of the heart failure epidemic. JAMA.

[24] Ceia F., Fonseca C., Mota T., Morais H., Matias F., De Sousa A., Oliveira A., EPICA Investigators (2002). Prevalence of chronic heart failure in Southwestern Europe: The EPICA study. Eur. J. Heart Fail..

[25] Quality and Outcomes Framework (QOF) 2015–2016. http://medical.cdn.patient.co.uk.

[26] SIMG (Italian Society of General Medicine) Indicators and Quality Standards for Cardiovascular Pathologies in General Medicine. http://www.info.asl2abruzzo.it/files/140905_formazione-mmg_gestione-scompenso-cardiaco.pdf.

[27] Bungard T.J., McAlister F.A., Johnson J.A., Tsuyuki R.T. (2001). Underutilisation of ACE inhibitors in patients with congestive heart failure. Drugs.

[28] Marzluf B.A., Reichardt B., Neuhofer L.M., Kogler B., Wolzt M. (2015). Influence of drug adherence and medical care on heart failure outcome in the primary care setting in Austria. Pharmacoepidemiol. Drug Saf..

[29] Cancian M., Battaggia A., Celebrano M., Del Zotti F., Novelletto B.F., Michieli R., Saugo M., Pellizzari M., Toffanin R. (2013). The care for chronic heart failure by general practitioners. Results from a clinical audit in Italy. Eur. J. Gen. Pract..

[30] Buja A., Gini R., Visca M., Damiani G., Federico B., Donato D., Francesconi P., Marini A., Donatini A., Brugaletta S. (2014). Need and disparities in primary care management of patients with diabetes. BMC Endocr. Disord..

[31] WHO 2008–2013 Action Plan for the Global Strategy for Prevention and Control of Noncommunicable Disease. http://www.who.int/nmh/Actionplan-PC-NCD-2008.

[32] Doran T., Fullwood C., Kontopantelis E., Reeves D. (2008). Effect of financial incentives on inequalities in the delivery of primary clinical care in England: Analysis of clinical activity indicators for the quality and outcomes framework. Lancet.

[33] Piepoli M.F., Villani G.Q., Aschieri D., Bennati S., Groppi F., Pisati M.S., Rosi A., Capucci A. (2006). Multidisciplinary and multisetting team management programme in heart failure patients affects hospitalisation and costing. Int. J. Cardiol..

